# Segmentation and differentiation of periventricular and deep white matter hyperintensities in 2D T2-FLAIR MRI based on a cascade U-net

**DOI:** 10.3389/fneur.2022.1021477

**Published:** 2022-11-17

**Authors:** Tan Gong, Hualu Han, Zheng Tan, Zihan Ning, Huiyu Qiao, Miaoxin Yu, Xihai Zhao, Xiaoying Tang, Gaifen Liu, Fei Shang, Shuai Liu

**Affiliations:** ^1^Department of Biomedical Engineering, Beijing Institute of Technology School of Life Science, Beijing, China; ^2^Department of Biomedical Engineering, Center for Biomedical Imaging Research, Tsinghua University School of Medicine, Beijing, China; ^3^Department of Neurology, Beijing Tiantan Hospital, Capital Medical University, Beijing, China; ^4^China National Clinical Research Center for Neurological Diseases, Beijing, China

**Keywords:** periventricular white matter hyperintensities, deep white matter hyperintensities, image segmentation, cascade U-net, 2D T2-FLAIR

## Abstract

**Background:**

White matter hyperintensities (WMHs) are a subtype of cerebral small vessel disease and can be divided into periventricular WMHs (pvWMHs) and deep WMHs (dWMHs). pvWMHs and dWMHs were proved to be determined by different etiologies. This study aimed to develop a 2D Cascade U-net (Cascade U) for the segmentation and differentiation of pvWMHs and dWMHs on 2D T2-FLAIR images.

**Methods:**

A total of 253 subjects were recruited in the present study. All subjects underwent 2D T2-FLAIR scan on a 3.0 Tesla MR scanner. Both contours of pvWMHs and dWMHs were manually delineated by the observers and considered as the gold standard. Fazekas scale was used to evaluate the burdens of pvWMHs and dWMHs, respectively. Cascade U consisted of a segmentation U-net and a differentiation U-net and was trained with a combined loss function. The performance of Cascade U was compared with two other U-net models (Pipeline U and Separate U). Dice similarity coefficient (DSC), Matthews correlation coefficient (MCC), precision, and recall were used to evaluate the performances of all models. The linear correlations between WMHs volume (WMHV) measured by all models and the gold standard were also conducted.

**Results:**

Compared with other models, Cascade U exhibited a better performance on WMHs segmentation and pvWMHs identification. Cascade U achieved DSC values of 0.605 ± 0.135, 0.517 ± 0.263, and 0.510 ± 0.241 and MCC values of 0.617 ± 0.122, 0.526 ± 0.263, and 0.522 ± 0.243 on the segmentation of total WMHs, pvWMHs, and dWMHs, respectively. Cascade U exhibited strong correlations with the gold standard on measuring WMHV (R^2^ = 0.954, *p* < 0.001), pvWMHV (R^2^ = 0.933, *p* < 0.001), and dWMHV (R^2^ = 0.918, *p* < 0.001). A significant correlation was found on lesion volume between Cascade U and gold standard (r > 0.510, *p* < 0.001).

**Conclusion:**

Cascade U showed competitive results in segmentation and differentiation of pvWMHs and dWMHs on 2D T2-FLAIR images, indicating potential feasibility in precisely evaluating the burdens of WMHs.

## Introduction

Cerebral small vessel disease (CSVD) is characterized as a group of pathological processes with various etiologies affecting small arteries, arterioles, venules, and capillaries of the brain (1). White matter hyperintensities (WMHs) are commonly observed MRI-based biomarkers for CSVD. Some studies have validated that WMHs are closely related to an increased risk of stroke (2, 3), and higher WMHs load is observed in patients with depression (4), Alzheimer's disease (AD) (5), and migraine (6). WMHs, in view of the location, can be further divided into periventricular WMHs (pvWMHs) which extend from the ventricular wall and deep WMHs (dWMHs) in deep white matter area. pvWMHs and dWMHs show distinct risk factors and clinical implications. pvWMHs are associated with a decline in cerebral blood flow and cognitive function, and dWMHs are of hypoxic/ischemic origin and linked with a higher incidence of migraine and mood disorder (7, 8). pvWMHs-dWMHs dichotomization plays an important role in potential therapeutic intervention. The changes on fluidity and water content of interstitial tissue fluid in patient with WMHs may be reversible if intervention is conducted at early stage (9).

Magnetic resonance imaging T2-weighted fluid-attenuated inversion recovery (T2-FLAIR) has been widely used to evaluate WMHs. On T2-FLAIR images, WMHs appear as hyperintense objects scattered throughout the white matter and cerebrospinal fluid is nullified for enhanced discrimination of ischemic pathology (10). Traditionally, qualitative and quantitative evaluations of WHMs relied on the radiologists' subjective scale or manual delineations, which were time-consuming and laborious (11). Convolutional neural network (CNN) has been validated as an efficient tool for the automatic segmentation of WMHs (12–18). A fine-tuned fully convolutional network (FCN) which combined linearly fine to coarse feature maps of a pretrained Visual Geometry Group was designed for the automatic segmentation of WMHs (12). Wang et al. developed an FCN structure that consisted of three U-shaped networks to segment WMHs using different shapes of patch (13). Wu et al. segmented WMHs on skull-stripped images using a skip connection U-net which could capture more features and speed the optimization convergence (14). Li et al. proposed an ensemble architecture that generated the segmentation of WMHs from multiple U-net models and the average of which was taken as the prediction result (15). In addition, two-dimensional (2D) U-net had the potential to distinguish WMHs from acute ischemic lesions (16). Recently, precise segmentations of pvWMHs and dWMHs attracted investigators' attention. An ensemble network architecture (TrUE-Net) of three parallel U-net using coronal, sagittal, and horizontal planes as independent input was proposed to segment and differentiate pvWMHs and dWMHs (17). Mojiri et al. presented a U-shaped three-dimensional (3D) Bayesian network to segment WMHs and a secondary 3D U-net to differentiate pvWMHs and dWMHs (18). However, these networks were designed for 3D thin-section scan. 2D T2-FLAIR was recommended in CSVD studies and acquired with high efficiency in community study (19, 20). Previous studies validated that 3D networks were more susceptible to limited axial slices compared with 2D networks (21).

Previous studies exhibited potential of cascaded CNN in WMHs segmentation on 3D T2-FLAIR images. However, the segmentation of small WMHs lesions on 2D FLAIR images was still a challenge, and further research was needed for accurate segmentation and differentiation of pvWMHs and dWMHs (13, 16). In this study, a cascade CNN (Cascade U) with a combined loss function was developed to segment and differentiate small pvWMHs and dWMHs on 2D T2-FLAIR images.

## Materials

### Dataset

In the present study, all subjects were recruited from a community study of Cardio- and cerebrovascular Accident Monitoring, Epidemiology, and caRe quAlity system (CAMERA) (22). A total of 253 subjects (30–80 years old, 111 male patients) were included from January 2017 to August 2020. The breakdown of subjects in our study is presented in Table 1. Each subject underwent 2D T2-FLAIR scan (TR/TE = 7000 ms/140 ms, flip angle = 90°, FOV = 230×230×133 mm^3^, voxel size = 0.9×0.9×5.5 mm^3^, matrix size = 256×256) on a 3.0 Tesla MR scanner (Achieva TX, Philips Healthcare, Best, The Netherlands) with a 32-channel phase array head coil. Repeat scan was conducted if the image quality was poor by visual assessment with the following criteria: (I) contrast-based: unclear or invisible contrast in gray matter and white matter; and (II) artifact-based: severe head motion, signal drop, or geometric distortion.

**Table 1:** Clinical characteristics of the study population.

	**All subjects**	**Train set**	**Test set**	**P**
	**(*n* = 253)**	**(*n* = 176)**	**(*n* = 77)**	
Age (year)	57.5 ± 12.7	56.5 ± 12.4	59.8 ± 13.2	0.067
Sex (male)	111 (43.9%)	80 (45.5%)	31 (40.3%)	0.444
BMI (kg/m^2^)	24.3 ± 3.1	24.4 ± 3.0	24.1 ± 3.4	0.415
Hypertension	80 (31.5%)	59 (33.5%)	21 (27.3%)	0.325
Hyperlipidemia	98 (38.7%)	66 (37.5%)	32 (41.6%)	0.542
Diabetes	30 (11.9%)	22 (12.5%)	8 (10.4%)	0.886
Smoking ^a^	31 (17.6%)	23 (18.5%)	8 (15.4%)	0.615
Volume (ml)				
WMHV	1.53 (0.58–3.60)	1.42 (0.57–3.37)	1.90 (0.71–4.00)	0.328
pvWMHV	0.82 (0.31–1.87)	0.82 (0.32–1.75)	0.86 (0.29–2.19)	0.946
dWMHV	0.44 (0.11–1.75)	0.41 (0.10–1.57)	0.73 (0.20–2.05)	0.094

Values are provided as mean ± standard deviation, median (interquartile range), or numbers (%) for each variable. BMI, body mass index; WMHV, white matter hyperintensity; pvWMHV, periventricular white matter hyperintensity volume; dWMHs, deep white matter hyperintensity.

a Subjects with unknown smoking status (52 in train set and 25 in test set) were excluded.

### Gold standard

**Figure 1:**
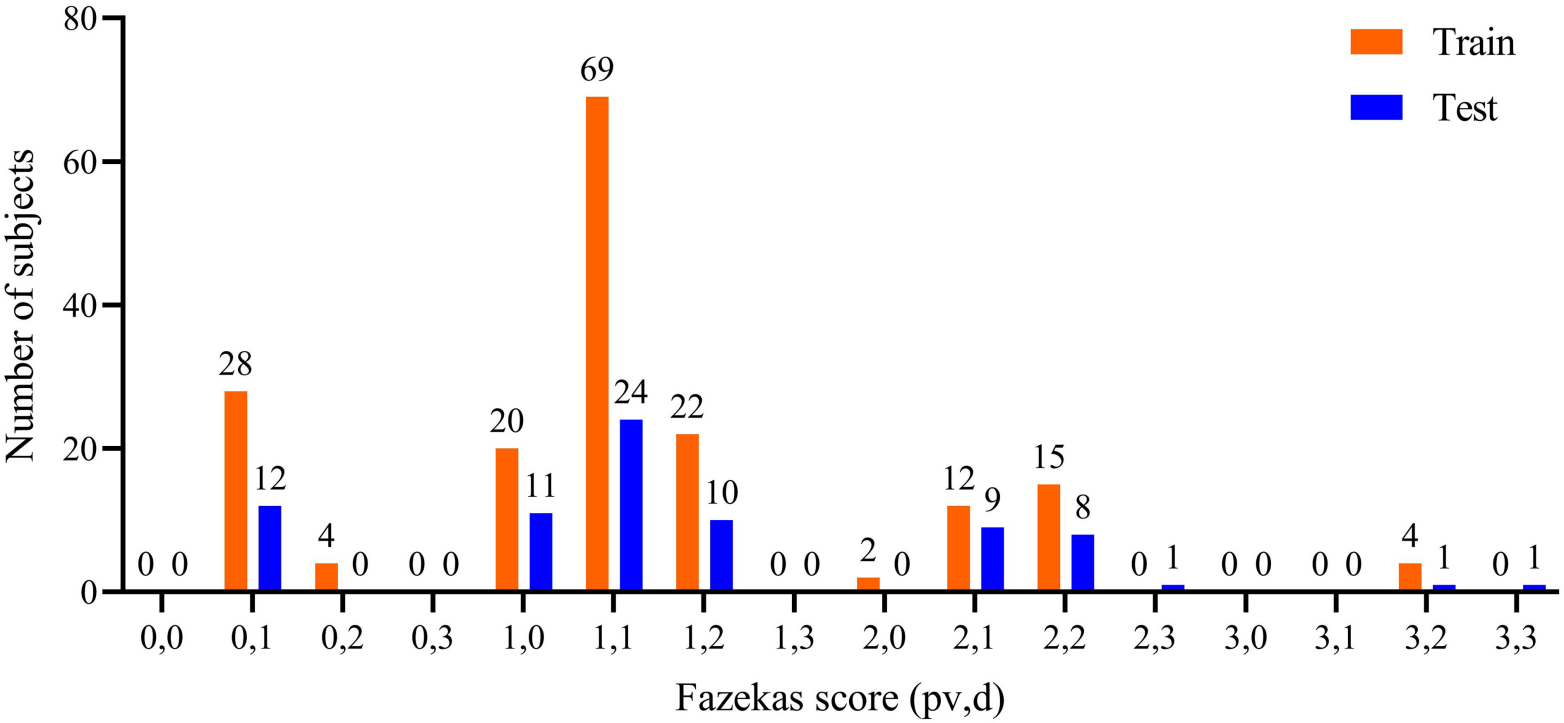
Distribution of pvWMHs and dWMHs Fazekas scores. pv represented Fazekas scores of pvWMHs, and d represented the Fazekas scores of dWMHs.

All T2-FLAIR images were reviewed by two radiologists with > 3 years' experience in neuroradiology with consensus. The contour delineated by the observer was considered as the gold standard. Each slice was interpolated from 256×256 to 512×512 for lesion delineation. In addition, Fazekas scales (ranging from 0 to 3 for pvWMHs and dWMHs, respectively) were used for describing the degree of WMHs (23). The distribution of Fazekas scales is shown in Figure 1. Ninety % of the subjects (223/253) were with a total Fazekas score lower than 3 (range 0 to 6).

## Methods

### Network architecture

In the present study, 2D CNNs were applied due to a voxel size of 5.5 mm along the vertical axis and small lesions. The cascade U-net architecture (Cascade U) was constructed based on a U-net architecture proposed by Ronneberger et al. in 2015 (24). Two other models (Pipeline U-net and Separate U-net) were trained as comparison.

**Figure 2:**
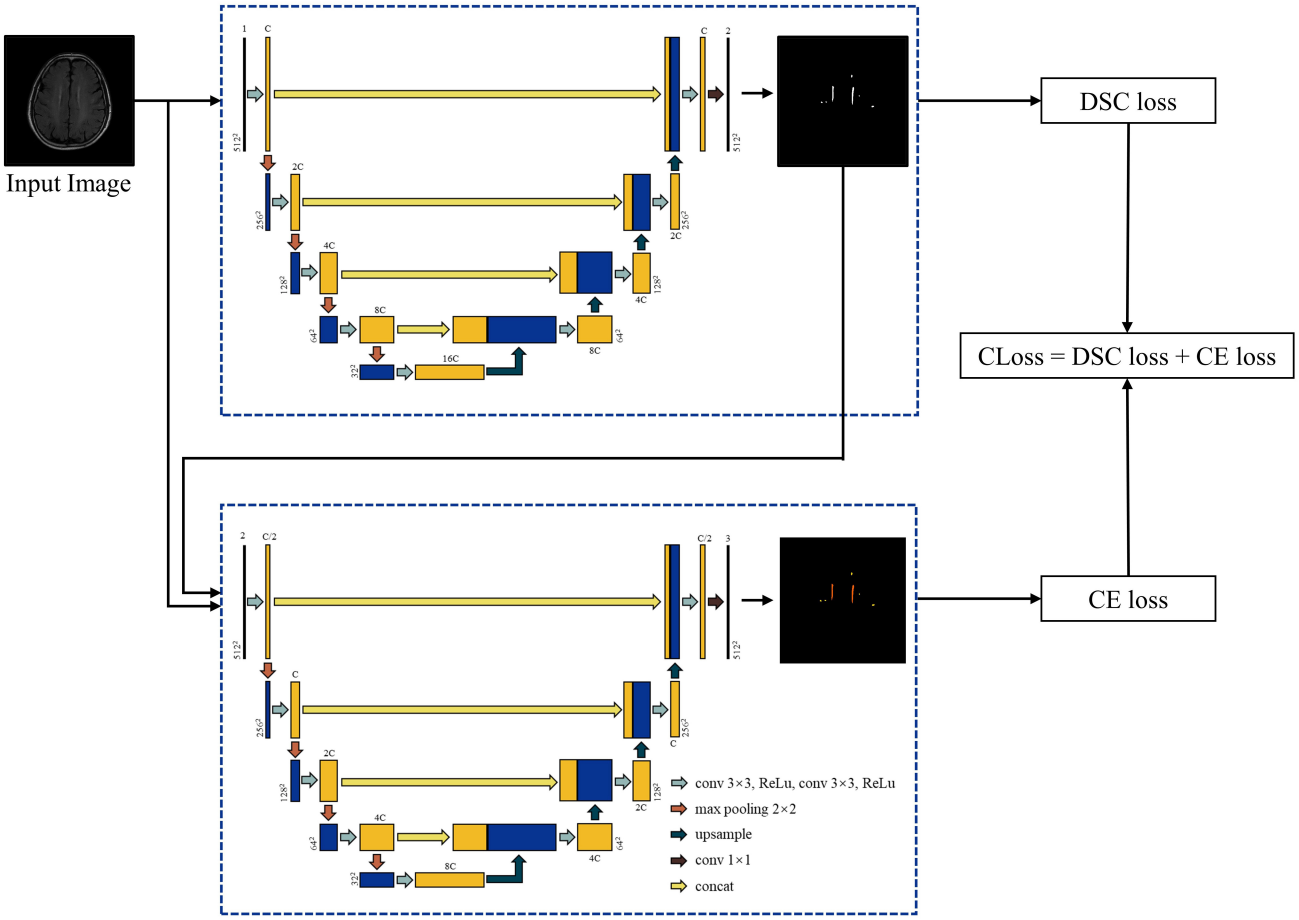
Architecture of Cascade U model. C represented the channel number of Cascade U model in the first layer and was selected as 64 in the present study.

a) Cascade U-net: Cascade U was integrated by segmentation stage and differentiation stage, and trained with a combined loss function (CLoss). The architecture is exhibited in Figure 2. In the first stage, the input was each T2-FLAIR slice, and the result was the WMHs segmentation result. In the second stage, pvWMHs and dWMHs were differentiated using the original slice and possibility map of WMHs segmentation from the first stage. The CLoss was defined by a Dice similarity (DSC) loss from the segmentation stage and cross-entropy (CE) loss from the differentiation stage. The loss functions were defined as follows:


$$\begin{array}{lll} CLoss\; & = & \;DSC\;Loss+CE\;Loss\\ DSC\;Loss\; & = & \;1-\frac{2\sum_{n=1}^{N}p_{n}g_{n}+\varepsilon }{\sum_{n=1}^{N}\left(p_{n}+g_{n}\right)+\varepsilon }\\ CE\;Loss\; & = & \;-\frac{1}{N}\overset{N}{\underset{n=1}{\sum }}\overset{Class}{\underset{i=1}{\sum }}y_{ni}\log{\hat{y}}_{ni}\; \end{array}$$

where *N* represented the number of pixels of the input data in both CE loss and DSC loss. In DSC loss, *p*_*n*_ represented the softmax output probability of WMHs at the *n*th pixel, *g*_n_ = 0 or 1 represented the ground truth of the *n*th pixel. The ε term was 10^−4^ to prevent the denominator being zero. In CE loss, *Class* represented the number of classes, ŷ_*ni*_ represented the softmax output probability of class *i* at the *n*th pixel, and *y*_*ni*_ represented the given one-hot encoded label for class *i* of the *n*th pixel.

b) Pipeline U-net: Pipeline U-net (Pipeline U) consisted of two U-net models. The first model was trained to segment WMHs with the addition of DSC loss and CE loss, while the second model was trained to differentiate pvWMHs and dWMHs with CE loss. Two models were trained independently.

c) Separate U-net: Separate U-net (Separate U) was two U-net models. Two models were trained to segment pvWMHs and dWMHs with the addition of DSC loss and CE loss, respectively.

The segmentation stages in Cascade U and Pipeline U were same, while the differentiation stages in three networks were same. All three models are publicly available at https://github.com/GGTTGTGT-2020/Cascade-U-Net-white-matter-hyperintensities-.

### Model training

A total of 176 subjects were randomly selected as training set (18 subjects for validation), and 77 subjects were testing set. Considering CNN's ability to segment brain MR images without preprocessing (25), we only conducted a Z-score normalization.

Data augmentation is an effective method to improve the robustness of models and the precision of prediction results. Five transformations were applied on training set for each slice in every epoch: 1) rotating: [−30°, 30°], 2) shifting: [−26 pixels, 26 pixels], 3) scaling: [0.9, 1.1], 4) horizontal flipping, and 5) changing the intensity of each pixel according to the following formula:


$${\left(\frac{x-min(x)}{max(x)-min(x)}\right)}^{\gamma }\times (max(x)-min(x))+min(x)$$

where γ was randomly selected from [0.5, 1]. The segmentation stage in Cascade U was pretrained for 200 epochs with WMHs gold standard on training set to accelerate convergence speed.

Each model was convergent after being trained for 500 epochs, and the model with the minimal loss on validation set was taken as the prediction model. Batch size was set to 6. Adam optimizer was used with an initial learning rate of 2×10^−3^ (26). The models were trained on an RTX 3090 graphics card with 24G of memory, and all models were constructed based on PyTorch 1.11.0 (27).

### Evaluation metrics

DSC, Matthews correlation coefficient (MCC), recall, precision, and the correlation coefficient of lesion volume were used to evaluate the performance of the models.

The DSC is defined as follows:


$$\begin{array}{l} DSC\;=\;2\times \frac{\left|G\cap P\right|}{\left|G|+|P\right|}\; \end{array}$$

where *P* represented a binary mask, and *G* represented the gold standard.

The MCC is an index to measure the quality of a binary classification system when the size of samples in the two classes varies substantially (28). The MCC is defined as follows:


$$MCC\;=\frac{TP\times TN-FP\times FN}{\sqrt{\left(TP+FP)\times (TP+FN)\times (TN+FP)\times (TN+FN\right)}}$$

where *TP, TN, FP*, and *FN* represented true positive, true negative, false positive, and false negative, respectively. The range of MCC is [−1, 1]. A value of 1 means that the prediction is completely consistent with actual result, 0 means not as good as random prediction result, and −1 means completely inconsistent with actual result.

The recall for lesions is defined as follows:


$$\begin{array}{l} Recall\;=\;\frac{TP}{TP+FN}\; \end{array}$$

The precision for lesions is defined as follows:


$$\begin{array}{l} Precision\;=\;\frac{TP}{TP+FP}\; \end{array}$$

Linear correlation analysis between WMH volumes (WMHVs) measured by three models and the gold standard was also employed to evaluate the performance of all models. The non-parametric Mann–Whitney U-test was conducted to assess the performance between Cascade U and other models. To test the association between clinical characteristics and the segmentation performance of Cascade U, the Mann–Whitney U-test and Spearman's correlation analysis were conducted on binary and continuous variables, respectively. A *p* < 0.05 was considered statistically significant, and all statistical analyses were conducted on SPSS v25.0 (International Business Machines, Inc., New York, USA). Continuous variables were presented as mean ± standard deviation (SD).

## Results

**Figure 3:**
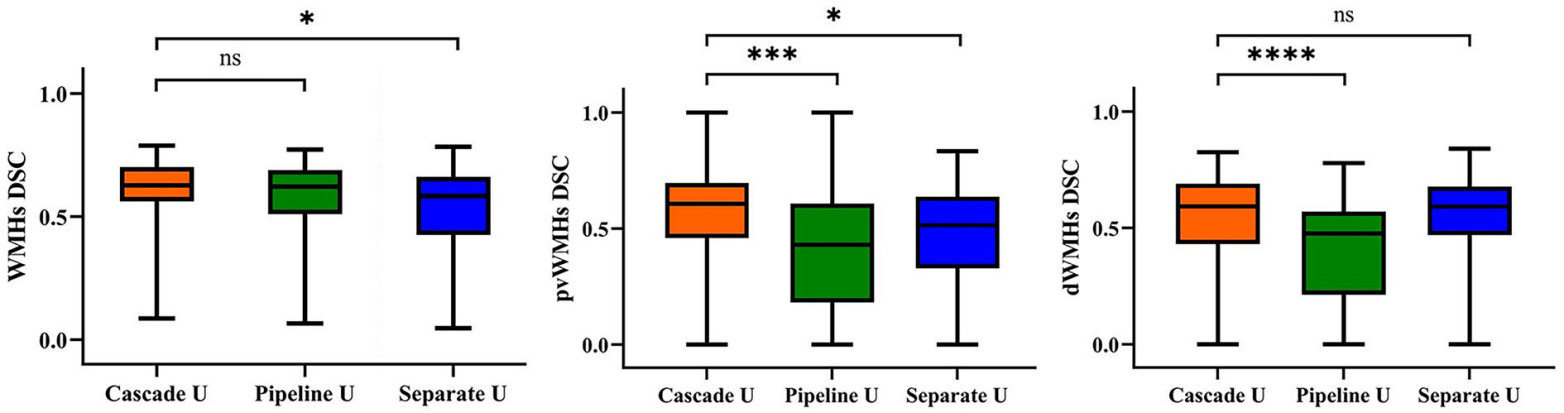
Statistical evaluation of WMHs, pvWMHs, and dWMHs segmentations using DSC. Not significant: ns, *p* < 0.05: ^*^; *p* < 0.001: ^***^; *p* < 0.0001: ^****^.

The mean values of DSC, MCC, precision, and recall for Cascade U, Pipeline U, and Separate U are summarized in Table 2. Cascade U had the highest DSC and MCC on WMHs segmentation and pvWMHs among the three models. On dWMHs segmentation, DSC and MCC of Cascade U were similar with those of Separate U. Compared with other two models, Cascade U exhibited the highest precision. However, Separate U produced the highest recall. Figure 3 shows the comparison between Cascade U and other models on DSC of WMHs, pvWMHs, and dWMHs segmentation.

**Table 2:** Performance of three models on total WMHs, pvWMHs, and dWMHs segmentations.

	**Total WMHs**	**pvWMHs**	**dWMHs**
**Cascade U**			
DSC	0.605 ± 0.135	0.517 ± 0.263	0.510 ± 0.241
MCC	0.617 ± 0.122	0.526 ± 0.263	0.522 ± 0.243
Precision	0.641 ± 0.184	0.551 ± 0.282	0.588 ± 0.293
Recall	0.621 ± 0.131	0.679 ± 0.223	0.626 ± 0.214
**Pipeline U**			
DSC	0.593 ± 0.135	0.389 ± 0.254	0.405 ± 0.218
MCC	0.607 ± 0.120	0.403 ± 0.254	0.421 ± 0.215
Precision	0.593 ± 0.181	0.476 ± 0.284	0.398 ± 0.242
Recall	0.655 ± 0.137	0.531 ± 0.298	0.629 ± 0.222
**Separate U**			
DSC	0.540 ± 0.171	0.451 ± 0.247	0.513 ± 0.240
MCC	0.563 ± 0.147	0.469 ± 0.245	0.523 ± 0.239
Precision	0.501 ± 0.219	0.401 ± 0.247	0.515 ± 0.261
Recall	0.687 ± 0.132	0.736 ± 0.192	0.698 ± 0.193

DSC, dice similarity coefficient; MCC, Matthews correlation coefficient.

The performance of all models on different levels of Fazekas scores is summarized in Tables 3 and 4. On pvWMHs segmentation (Table 3), Cascade U achieved the highest DSC, MCC, and precision on subjects in all levels, but lower recall in subjects with Fazekas scores of 1 and 2 than Separate U. On dWMHs segmentation (Table 4), Separate U showed a similar performance with Cascade U on the cases with a Fazekas score < 3, but the highest DSC, MCC, precision, and recall on the cases with a Fazekas score of 3. The association between demographic, clinical factors, and the segmentation results of Cascade U is summarized in Table 5. A significant difference was found on dWMHs segmentation results between the hypertensive and non-hypertensive groups. In addition, there was a significant correlation between segmentation results and lesion volume.

**Table 3:** Performance of three models on subjects' pvWMHs with different Fazekas scores.

**Fazekas scores**	**pvWMHs**			
	**0 (*n* = 12)**	**1 (*n* = 45)**	**2 (*n* = 18)**	**3 (*n* = 2)**
**Cascade U**				
DSC	0.083 ± 0.276	0.562 ± 0.174	0.672 ± 0.099	0.729 ± 0.067
MCC	0.083 ± 0.276	0.575 ± 0.169	0.677 ± 0.091	0.740 ± 0.058
Precision	0.083 ± 0.276	0.624 ± 0.194	0.644 ± 0.116	0.865 ± 0.017
Recall	1.000 ± 0.000	0.578 ± 0.207	0.720 ± 0.094	0.640 ± 0.111
**Pipeline U**				
DSC	0.083 ± 0.276	0.397 ± 0.212	0.556 ± 0.127	0.516 ± 0.185
MCC	0.083 ± 0.276	0.415 ± 0.210	0.564 ± 0.125	0.578 ± 0.153
Precision	0.083 ± 0.276	0.539 ± 0.239	0.550 ± 0.151	0.773 ± 0.004
Recall	1.000 ± 0.000	0.383 ± 0.242	0.594 ± 0.151	0.466 ± 0.230
**Separate U**				
DSC	0.000 ± 0.000	0.487 ± 0.168	0.635 ± 0.006	0.677 ± 0.094
MCC	0.000 ± 0.000	0.515 ± 0.154	0.643 ± 0.005	0.696 ± 0.075
Precision	0.000 ± 0.000	0.420 ± 0.188	0.573 ± 0.006	0.849 ± 0.045
Recall	1.000 ± 0.000	0.674 ± 0.184	0.730 ± 0.006	0.587 ± 0.154

DSC, dice similarity coefficient; MCC, Matthews correlation coefficient.

**Table 4:** Performance of three models on subjects' dWMHs with different Fazekas scores.

**Fazekas scores**	**dWMHs**			
	**0 (*n* = 12)**	**1 (*n* = 44)**	**2 (*n* = 19)**	**3 (*n* = 2)**
**Cascade U**				
DSC	0.000 ± 0.000	0.567 ± 0.142	0.661 ± 0.070	0.583 ± 0.113
MCC	0.000 ± 0.000	0.583 ± 0.136	0.674 ± 0.063	0.584 ± 0.112
Precision	0.000 ± 0.000	0.646 ± 0.194	0.792 ± 0.077	0.584 ± 0.151
Recall	1.000 ± 0.000	0.555 ± 0.178	0.584 ± 0.122	0.587 ± 0.073
**Pipeline U**				
DSC	0.000 ± 0.000	0.440 ± 0.158	0.563 ± 0.098	0.343 ± 0.140
MCC	0.000 ± 0.000	0.464 ± 0.142	0.572 ± 0.093	0.352 ± 0.144
Precision	0.000 ± 0.000	0.392 ± 0.177	0.635 ± 0.107	0.452 ± 0.186
Recall	1.000 ± 0.000	0.597 ± 0.175	0.527 ± 0.140	0.276 ± 0.112
**Separate U**				
DSC	0.000 ± 0.000	0.570 ± 0.138	0.663 ± 0.069	0.629 ± 0.058
MCC	0.000 ± 0.000	0.585 ± 0.126	0.669 ± 0.068	0.628 ± 0.057
Precision	0.000 ± 0.000	0.550 ± 0.171	0.718 ± 0.089	0.646 ± 0.044
Recall	1.000 ± 0.000	0.656 ± 0.176	0.633 ± 0.121	0.613 ± 0.070

DSC, dice similarity coefficient; MCC, Matthews correlation coefficient.

**Table 5:** Association between demographic, clinical factors, and the segmentation results of Cascade U on testing set.

	**Total WMHs**		**pvWMHs**		**dWMHs**	
**BV**	**DSC**	**P**	**DSC**	**P**	**DSC**	**P**
Hypertension (yes = 1)	0.601 ± 0.134	0.575	0.557 ± 0.232	0.211	0.473 ± 0.255	0.010
	0.617 ± 0.146		0.410 ± 0.103		0.608 ± 0.179	
Hyperlipidemia (yes = 1)	0.600 ± 0.130	0.397	0.488 ± 0.263	0.125	0.539 ± 0.226	0.222
	0.612 ± 0.146		0.559 ± 0.267		0.468 ± 0.263	
Diabetes (yes = 1)	0.600 ± 0.141	0.473	0.501 ± 0.275	0.160	0.510 ± 0.247	0.536
	0.648 ± 0.082		0.655 ± 0.078		0.505 ± 0.220	
Smoking ^a^ (yes = 1)	0.596 ± 0.151	0.389	0.535 ± 0.236	0.576	0.469 ± 0.266	0.909
	0.564 ± 0.131		0.429 ± 0.153		0.490 ± 0.229	
Sex (male)	0.610 ± 0.149	0.355	0.512 ± 0.257	0.399	0.512 ± 0.243	0.763
	0.598 ± 0.116		0.481 ± 0.277		0.507 ± 0.247	
CV						
BMI	r = −0.032	0.783	r = −0.075	0.525	r = 0.032	0.787
Age	r = −0.109	0.346	r = −0.079	0.492	r = 0.150	0.194
Lesion volume	r = 0.689	<0.001	r = 0.557	<0.001	r = 0.510	<0.001

Values are provided as mean ± standard deviation. BV, binary variables; CV, continuous variables; DSC, dice similarity coefficient; BMI, body mass index.

a Subjects with unknown smoking status (25 in testing set) were excluded.

**Figure 4:**
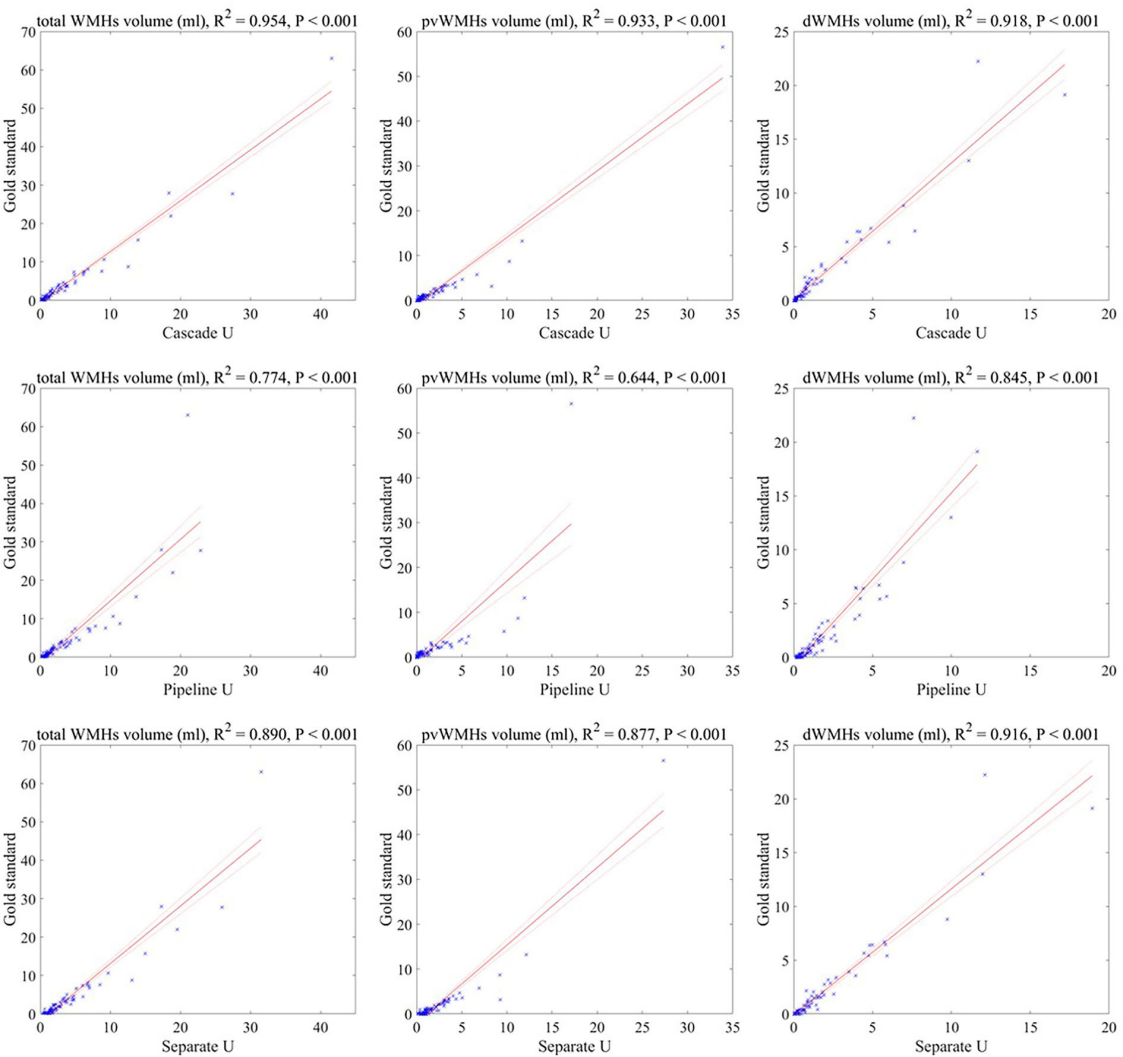
Correlations between the lesion volumes measured by three models and the gold standard. Solid line represented fitting result, and dashed line represented 95% confidence interval.

The correlations between WMHV measured by radiologists and by three models are summarized in Figure 4. Cascade U model had the highest R^2^ in WMHV (R^2^ = 0.954, *p* < 0.001), pvWMHV (R^2^ = 0.933, *p* < 0.001), and dWMHV (R^2^ = 0.918, *p* < 0.001) among the three models.

## Discussion

In the present study, a 2D Cascade U model using a combined loss function was proposed to segment and identify pvWMHs and dWMHs simultaneously on T2-FLAIR images. Compared with Pipeline U and Separate U, Cascade U exhibited better performance on WMHs segmentation and pvWMHs identification. In addition, the lesion volume measured by Cascade U had the strongest correlation with the gold standard.

The cascade model exhibited advantages on many tasks, including segmentation of the pancreas (29, 30), brain tumor (31), and bladder cancer (32). Liu et al. developed two sub-models for the segmentation of WMHs and differentiation between focal cerebral ischemia and lacunar infarction (33). The results showed the superiority of the cascade model in the segmentation and differentiation of small brain lesions. In the present study, Cascade U outperformed Pipeline U and Separate U on the segmentation and identification of pvWMHs and dWMHs. Combined loss function made the learning process guided by the segmentation loss function (DSC) and classification loss function (CE) at the same time and improved model's performance.

**Figure 5:**
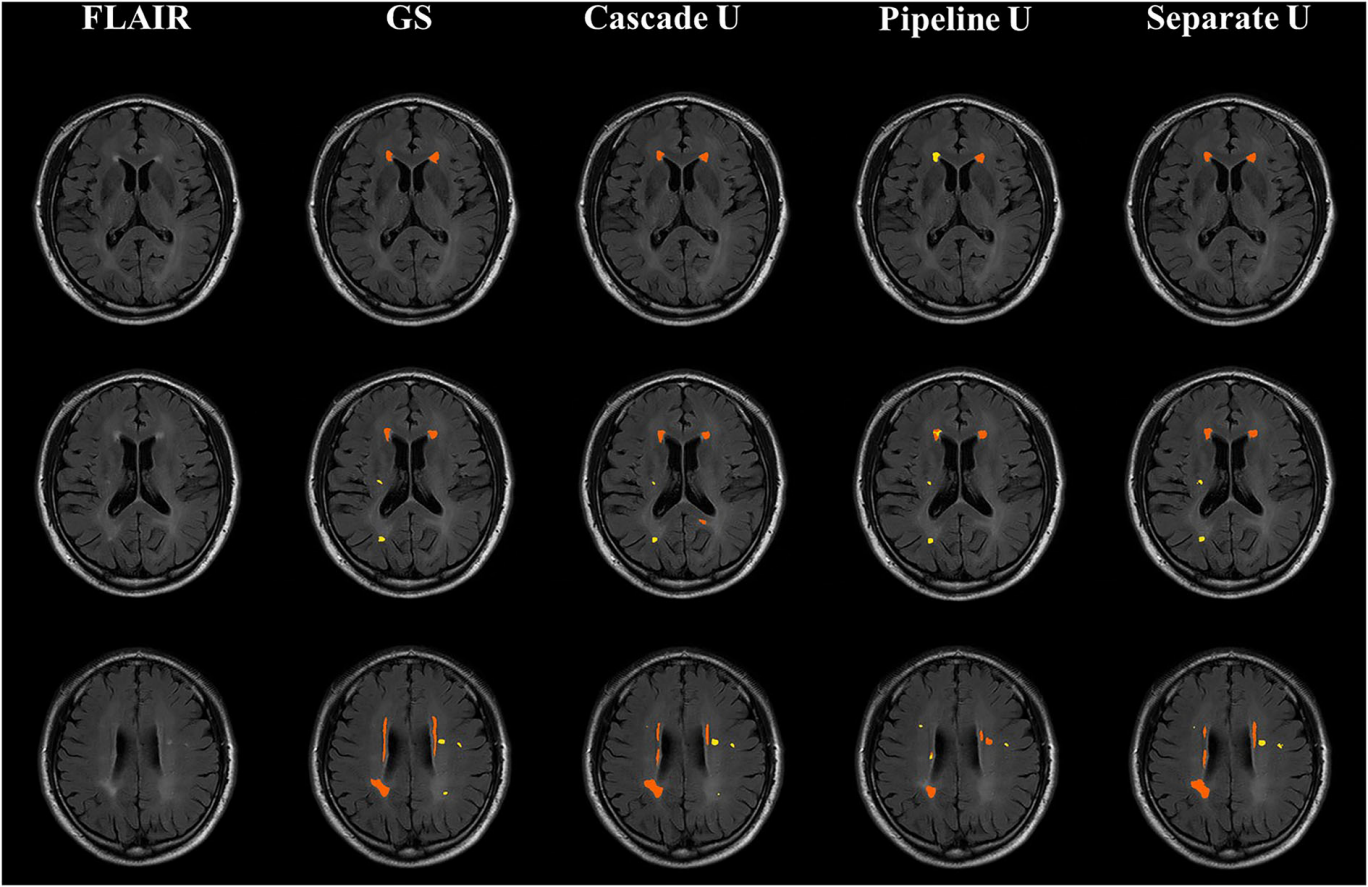
Illustration of segmentation results from a subject with Fazekas score of 3. From left to right were T2-FLAIR images, the gold standard, Cascade U prediction results, Pipeline U prediction results, and Separate U prediction results. Orange represented pvWMHs, and yellow represented dWMHs. GS, gold standard.

**Figure 6:**
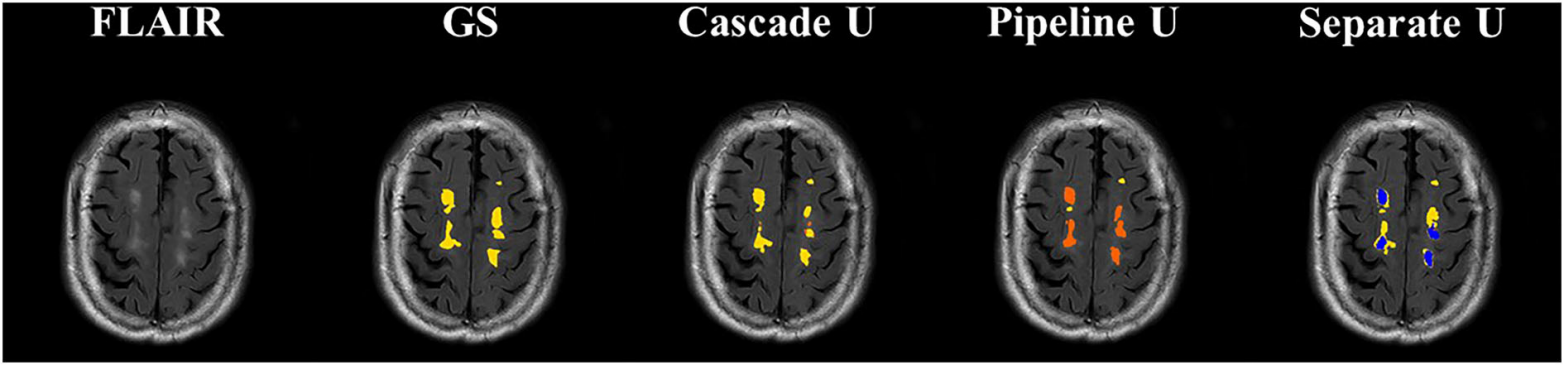
Illustration of segmentation results of dWMHs. Orange represented pvWMHs, yellow represented dWMHs, and blue represented the overlaps of the model prediction. GS, gold standard.

Figure 5 shows the comparisons between the gold standard and three models on the segmentation and differentiation of pvWMHs and dWHMs from a subject with a Fazekas score of 3 (pvWMHs: 2 and dWMHs: 1). Pipeline U underestimated and confused pvWMHs and dWMHs in some cases. In Pipeline U, the identification of pvWMHs and dWMHs relied on the prediction results of segmentation model, leading to the propagation of error. Cascade U overcame this problem *via* training segmentation network together with a differentiation network using a combined loss function. Compared with Cascade U, Separate U exhibited inferior performance on pvWMHs segmentation and similar performance on dWMHs segmentation. However, overlaps of pvWMHs and dWMHs were found in some cases (blue regions in Figure 6), exhibiting a region identified as pvWMHs and dWMHs simultaneously by two U-net models in Separate U. The example implied that independent training had some limitation in segmentation and differentiation of pvWMHs and dWMHs.

In this study, all subjects were recruited from community-based population aiming to investigate the risk factors of cerebrovascular disease risk. Early characterization of WMHs before cognitive symptoms occur may prevent further deterioration to dementia (34, 35). In the present study, 90% (223/253) of subjects recruited were at an early stage, with a total Fazekas score lower than 3 (range 0 to 6). The median of pvWMHV and dWMHV on each subject was 0.82 ml and 0.44 ml, respectively. Previous studies had validated that accurate segmentation of small WMHs was more challenging compared with large WMHs (13, 16, 33). Although exhibited potential in WMHs segmentation, CNN-based network was more likely to cause false negative prediction on the images with smaller lesions and thus led to a lower DSC (36). As exhibited in Table 3, the performance of each model on the segmentation and identification of pvWMHs and dWMHs increased significantly with the burden of WMHs. Our result also exhibited that the segmentation performance was correlated with lesion volume. In the present study, the subjects with hypertension exhibited a severer dWMHs burden compared with those without hypertension (1.71 ml vs. 0.60 ml), favoring the better segmentation performance on the hypertensive group than that on the non-hypertensive group.

**Figure 7:**
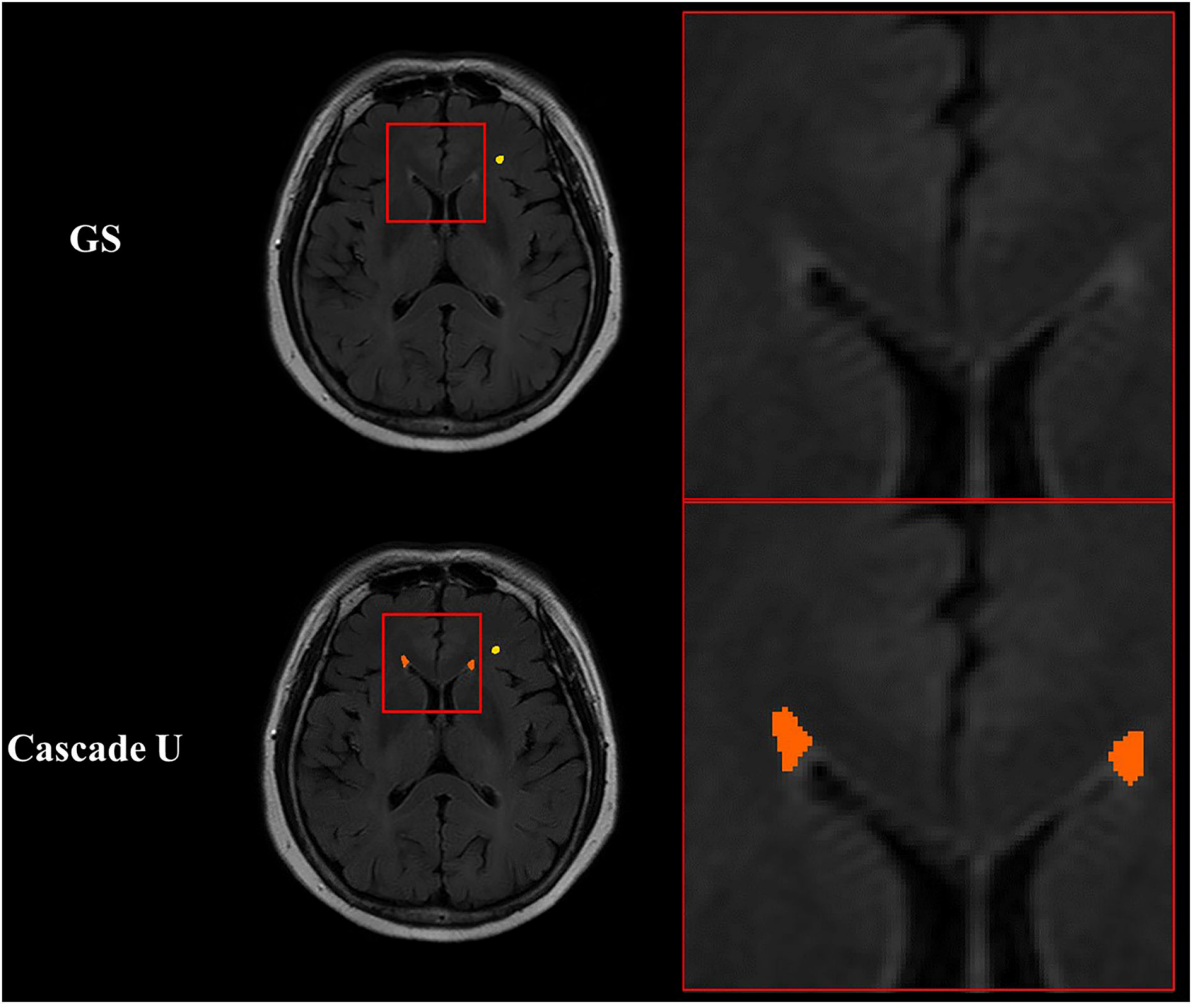
Illustration of segmentation results of Cascade U with pvWMHs Fazekas score of 0. Orange represented pvWMHs, and yellow represented dWMHs. GS, gold standard.

In addition, manual annotation was considered the gold standard, but it may not always reflect the real situation (17). It was more difficult to accurately delineate the contour of small lesions for radiologists due to low contrast between lesion and adjacent tissue or noise inherent in imaging protocol. Since DSC was a metric sensitivity to the size of object, the correlations between WMHs volume measured by three models and the gold standard were also compared in the present study. The strongest correlation between volume measured by Cascade U and the gold standard also demonstrated the advantage of Cascade U on the segmentation and differentiation of pvWMHs and dWMHs. Figure 7 exhibits some lesions detected by Cascade U on a subject with a pvWMHs score of 0, but missed in manual delineation by radiologists.

There were several limitations in the present study: 1) T1-weighted MRI can provide additional information to the segmentation of WMHs (37). Some subjects did not undergo T1-weighted MR scan, and only 2D T2-FLAIR images were used in the present study. In future, we will attempt to use multi-modality images, such as T1-weighted and diffusion-weighted imaging, as input of the model. 2) Cascade U was designed on a 2D U-net due to a layer thickness of 5.5 mm with 2D T2-FLAIR scan. 3D U-net can provide competitive results *via* leveraging spatial and anatomical information in volumetric organs (31). CNN with a 3D architecture would be investigated on the segmentation and differentiation of pvWMHs and dWMHs on 3D T2-FLAIR data. 3) Finally, our model was trained and tested on a single-center dataset. The models should be tested on multi-center datasets or on the crowds with different population characteristics in future.

## Conclusion

In this study, a cascade 2D U-net (Cascade U) was proposed for the segmentation and differentiation of pvWMHs and dWMHs. Cascade U was composed by a segmentation stage and a differentiation stage and trained with a combined loss function. Cascade U achieved better segmentation and differentiation of pvWMHs and dWMHs on 2D T2-FLAIR images and showed potential feasibility in precisely evaluating the burden of WMHs.

## Data availability statement

The original contributions presented in the study are included in the article/supplementary material, further inquiries can be directed to the corresponding author.

## Ethics statement

The studies involving human participants were reviewed and approved by Ethics Committee of Tsinghua University. The patients/participants provided their written informed consent to participate in this study.

## Author contributions

FS and SL designed the study. HH and HQ contributed to the data collection. XZ and GL contributed to the data management. HH, ZN, and MY interpreted the data. TG and ZT developed the network. TG, HH, and ZT performed the statistical analysis. TG, FS, and SL drafted the manuscript. SL, FS, HH, XT, and XZ revised the manuscript. All authors have read the manuscript and approved the submitted version.

## Funding

This study was funded by the grants of the National Natural Science Foundation of China (Nos. 82102136 and 81771825), the Beijing Municipal Commission of Health and Family Planning (No. 2016-1–2041), the Beijing Municipal Science and Technology Commission (Nos. D17110003017002 and D17110003017003), and the Ministry of Science and Technology of China (Nos. 2017YFC1307904, 2017YFC1307702, and 2016YFC0901001).

## Conflict of interest

The authors declare that the research was conducted in the absence of any commercial or financial relationships that could be construed as a potential conflict of interest.

## Publisher's note

All claims expressed in this article are solely those of the authors and do not necessarily represent those of their affiliated organizations, or those of the publisher, the editors and the reviewers. Any product that may be evaluated in this article, or claim that may be made by its manufacturer, is not guaranteed or endorsed by the publisher.
